# Supplementary material for: Advancing food security: Rice yield estimation framework using time-series satellite data & machine learning
Source: PLoS One. 2024 Dec 12;19(12):e0309982. doi: 10.1371/journal.pone.0309982 (PMC11637374; doi:10.1371/journal.pone.0309982)
Supplement: S1 Table — In the naming convention for MODIS bands and indices, the term ’Early’ pertained to the 15-day composite image representing the first half of the month (days 1–16), while ’Late’ pertained to the second half of the month (days 17–31). (DOCX) [file pone.0309982.s001.docx]

**Table S1: Random forest variable importance utilizing all input variables, including the MODIS bands, spectral indices, and land surface temperature. In the naming convention for MODIS bands and indices, the term 'Early' pertained to the 15-day composite image representing the first half of the month (days 1–16), while 'Late' pertained to the second half of the month (days 17–31)**

| Variable Importance | Value |
| --- | --- |
| srwi_Feb_Late | 0.072664 |
| srwi_Feb_Early | 0.05555 |
| lswi_Feb_Late | 0.040044 |
| lswi_Feb_Early | 0.030895 |
| sur_refl_b06_Feb_Early | 0.03012 |
| srwi_Jan_Late | 0.0266 |
| lswi_Jan_Late | 0.023934 |
| srwi_March_Early | 0.023647 |
| lswi_March_Early | 0.022984 |
| sur_refl_b06_Feb_Late | 0.020786 |
| sur_refl_b06_Jan_Late | 0.018454 |
| sur_refl_b05_Feb_Late | 0.016095 |
| savi_Jan_Early | 0.014472 |
| ndvi_Jan_Early | 0.013861 |
| ndsvi_Jan_Early | 0.012205 |
| ndvi_March_Early | 0.011433 |
| lswi_Jan_Early | 0.011416 |
| sur_refl_b07_Feb_Early | 0.01121 |
| sur_refl_b05_Feb_Early | 0.010739 |
| savi_March_Early | 0.010656 |
| sur_refl_b05_April_Late | 0.010338 |
| sur_refl_b07_Jan_Late | 0.010109 |
| sur_refl_b06_April_Late | 0.009739 |
| srti_Jan_Late | 0.009716 |
| srti_Jan_Early | 0.009212 |
| srwi_Jan_Early | 0.009172 |
| sur_refl_b02_April_Late | 0.008674 |
| sur_refl_b05_Jan_Late | 0.008567 |
| B7_Jan_Early | 0.008044 |
| ndvi_Jan_Late | 0.007886 |
| B2_Jan_Early | 0.007591 |
| savi_Feb_Early | 0.007586 |
| sur_refl_b06_March_Early | 0.007005 |
| sur_refl_b07_March_Early | 0.006983 |
| sur_refl_b07_Feb_Late | 0.006524 |
| ndvi_April_Early | 0.006482 |
| ndvi_April_Late | 0.006444 |
| B1_Jan_Early | 0.006207 |
| srti_March_Early | 0.006181 |
| savi_Jan_Late | 0.006075 |
| sur_refl_b07_April_Late | 0.006074 |
| sur_refl_b05_March_Early | 0.005821 |
| srwi_March_Late | 0.005799 |
| sur_refl_b01_March_Early | 0.005734 |
| sur_refl_b04_Jan_Late | 0.005602 |
| sur_refl_b02_April_Early | 0.005599 |
| savi_Feb_Late | 0.005586 |
| sur_refl_b03_April_Late | 0.005537 |
| ndsvi_March_Late | 0.0055 |
| mcrc_April_Early | 0.005477 |
| ndvi_March_Late | 0.00543 |
| sur_refl_b01_Feb_Late | 0.005418 |
| savi_March_Late | 0.005342 |
| ndsvi_Jan_Late | 0.005279 |
| ndti_Jan_Early | 0.005271 |
| ndti_Jan_Late | 0.005238 |
| savi_April_Late | 0.00522 |
| ndvi_Feb_Early | 0.005171 |
| ndti_March_Early | 0.005159 |
| savi_April_Early | 0.005121 |
| B4_Jan_Early | 0.005098 |
| sur_refl_b01_Jan_Late | 0.005059 |
| B6_Jan_Early | 0.004988 |
| sur_refl_b04_April_Late | 0.004834 |
| sur_refl_b02_Feb_Late | 0.004833 |
| sur_refl_b03_Jan_Late | 0.004821 |
| evi_March_Early | 0.004761 |
| evi_Jan_Late | 0.004577 |
| sur_refl_b01_April_Late | 0.004491 |
| B5_Jan_Early | 0.004333 |
| mcrc_Jan_Late | 0.00432 |
| B3_Jan_Early | 0.004299 |
| ndsvi_Feb_Early | 0.004245 |
| sur_refl_b02_Feb_Early | 0.004197 |
| sur_refl_b04_March_Late | 0.004169 |
| srti_Feb_Early | 0.004168 |
| crci_April_Early | 0.004155 |
| srti_April_Early | 0.004147 |
| sur_refl_b02_March_Early | 0.004063 |
| ndsvi_April_Early | 0.003926 |
| evi_April_Late | 0.003915 |
| sur_refl_b02_Jan_Late | 0.00383 |
| lswi_April_Late | 0.003748 |
| sur_refl_b03_March_Early | 0.003731 |
| evi_March_Late | 0.00373 |
| sur_refl_b05_April_Early | 0.003728 |
| sur_refl_b04_March_Early | 0.003724 |
| ndsvi_April_Late | 0.003718 |
| sur_refl_b03_Feb_Late | 0.003705 |
| mcrc_Feb_Early | 0.003624 |
| crci_Jan_Early | 0.003608 |
| ndsvi_March_Early | 0.003607 |
| sur_refl_b07_March_Late | 0.003582 |
| sur_refl_b04_Feb_Late | 0.003508 |
| sur_refl_b01_March_Late | 0.003461 |
| sur_refl_b06_March_Late | 0.003408 |
| mcrc_Jan_Early | 0.003386 |
| mcrc_Feb_Late | 0.003383 |
| srti_April_Late | 0.003281 |
| evi_Feb_Late | 0.003255 |
| sur_refl_b01_April_Early | 0.003247 |
| ndti_April_Late | 0.003192 |
| ndti_April_Early | 0.003179 |
| sur_refl_b07_April_Early | 0.00314 |
| srti_Feb_Late | 0.003074 |
| sur_refl_b03_April_Early | 0.003053 |
| sur_refl_b06_April_Early | 0.003046 |
| sur_refl_b05_March_Late | 0.003036 |
| sur_refl_b03_March_Late | 0.003013 |
| evi_April_Early | 0.002864 |
| sur_refl_b04_Feb_Early | 0.002857 |
| mcrc_March_Early | 0.002792 |
| srwi_April_Early | 0.002774 |
| crci_April_Late | 0.002751 |
| sur_refl_b02_March_Late | 0.002731 |
| sur_refl_b01_Feb_Early | 0.002664 |
| evi_Jan_Early | 0.002631 |
| srti_March_Late | 0.002629 |
| srwi_April_Late | 0.002608 |
| ndvi_Feb_Late | 0.002607 |
| sur_refl_b04_April_Early | 0.002589 |
| sur_refl_b03_Feb_Early | 0.002575 |
| lswi_March_Late | 0.002523 |
| evi_Feb_Early | 0.002515 |
| crci_Jan_Late | 0.00247 |
| crci_March_Late | 0.00237 |
| mcrc_April_Late | 0.00235 |
| ndti_Feb_Early | 0.002338 |
| mcrc_March_Late | 0.002272 |
| ndsvi_Feb_Late | 0.002145 |
| ndti_Feb_Late | 0.002074 |
| lswi_April_Early | 0.00199 |
| crci_Feb_Early | 0.001984 |
| crci_March_Early | 0.001687 |
| ndti_March_Late | 0.001588 |
| crci_Feb_Late | 0.001549 |
