# Supplementary material for: Advancing food security: Rice yield estimation framework using time-series satellite data & machine learning
Source: PLoS One. 2024 Dec 12;19(12):e0309982. doi: 10.1371/journal.pone.0309982 (PMC11637374; doi:10.1371/journal.pone.0309982)
Supplement: S2 Table — (DOCX) [file pone.0309982.s002.docx]

**S2 Table: Comparison between different validation methods in random forest classification.**

| Accuracy metrics | K-fold | Ratio of the dataset (70:30) | Leave-one-out | Held-one-year-out |
| --- | --- | --- | --- | --- |
| Average RMSE | 0.31 | 0.28 | 0.22 | 0.31 |
| Average R2 | 0.57 | 0.56 | 0.57 | 0.50 |
| Average MSE | 0.098 | 0.08 | 0.09 | 0.10 |
| Average percentage RMSE | 8.08 | 8.10 | 5.91 | 8.10 |
