# Supplementary material for: Advancing food security: Rice yield estimation framework using time-series satellite data & machine learning
Source: PLoS One. 2024 Dec 12;19(12):e0309982. doi: 10.1371/journal.pone.0309982 (PMC11637374; doi:10.1371/journal.pone.0309982)
Supplement: S1 Fig — a) magnitude of the modified mk test on district level rice yields, b) district level trends in the rice yields. (DOCX) [file pone.0309982.s003.docx]

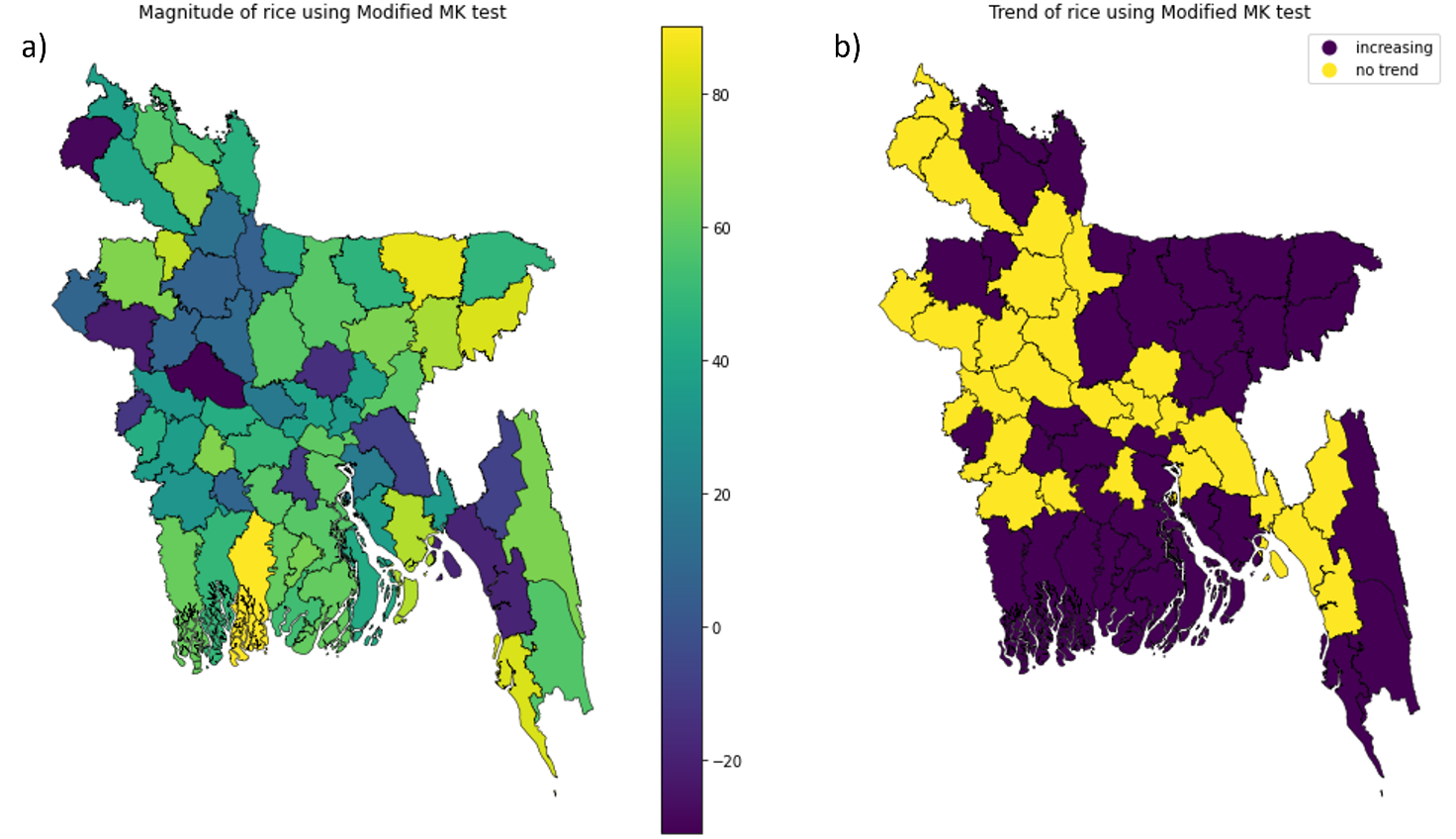


**S1 Fig: a) magnitude of the modified mk test on district level rice yields, b) district level trends in the rice yields**
