# Supplementary material for: Advancing food security: Rice yield estimation framework using time-series satellite data & machine learning
Source: PLoS One. 2024 Dec 12;19(12):e0309982. doi: 10.1371/journal.pone.0309982 (PMC11637374; doi:10.1371/journal.pone.0309982)
Supplement: Attachment — Submitted filename: Response_to_reviewers.docx [file pone.0309982.s004.docx]

**We would like to thank the reviewers for their valuable comments. Our responses are in Italics for each comment and the corresponding text is added in the track-changed and clean version of the manuscript.**

**Reviewer 1**

**1. Check for typos and blank spaces throughout the manuscript.**

*Thanks for bringing this up. We have thoroughly checked the spelling, grammar, and blank spaces throughout the manuscript.*

**2. Please ensure all figures are high quality. I encourage the authors to submit high-resolution versions of the figures for publication.**

*All the figures are generated per the journal guidelines (300 dpi).*

**3. The source of the shapefile used to create the figure is not mentioned in the text. Authors are encouraged to cite the source of the shapefile.**

**Modified line Lines 114-116, 127-130, Line 296-298, Line 309-313, Line 334-336:** *The shapefile reprinted from the GADM database under a CC BY license, with permission from Global Administrative Areas (www.gadm.org), original copyright 2018.*

*Added this statement in Fig1,2,7 and 8: “Shapefile reprinted from GADM database under a CC BY license, with permission from Global Administrative Areas (www.gadm.org), original copyright 2018. The figure was made with QGIS 3.22.7 under a CC BY license.”*

**4. 2.2.2. Reference data: It's unclear whether these yield data are for rice or all crops. Additionally, the timeframe for which the yields were collected is not mentioned.**

**We clarify that this was only rice yield data and added in Lines 136-139:** *“Additionally, crop yield data for rice were obtained through a collaborative effort involving the International Maize and Wheat Improvement Center (CIMMYT), the Bangladesh Agricultural Research Council (BARC), and the Bangladesh Rice Research Institute (BRRI) for boro rice crops”*

**5. 2.2.3. Satellite data: Here, you mention using spectral bands at 500-meter spatial resolution and LST data at 1000-meter resolution. It's unclear whether you resampled the MODIS spectral data to 1000 meters to match the LST resolution.**

**Revised the sentence in Lines 169-171** *“The MODIS surface reflectance bands and indices were resampled to a 1000-meter spatial resolution and stacked together with MODIS LST to derive seven surface reflectance bands, ten indices, and one LST band, equivalent to eighteen bands per composite.”*

**6. 2.3.1 Setting up, a random forest machine learning model: I'd recommend exploring additional cross-validation techniques like hold-one-year-out, leave-one-out, and 70:30 train-test splits. This can help assess the model's generalizability and consistency across different data partitions.**

Thank you for your suggestion, we did the additional analysis and found that the accuracy of the Random Forest regression method is not affected by the cross-validation technique. Here is the summary of our results.

*Line 207-208: “However, we also tested additional cross-validation techniques like hold-one-year-out, leave-one-out, and 70:30 train-test splits.”*

*Line 251-252: “We used a range of accuracy metrics when employing various input variables in the RF regression model with k-fold validation based on the Gini index ranking”*

*Line 269-271: “Additionally, we found that the accuracy of the Random Forest regression method is not affected by employing different cross-validation techniques (hold-one-year-out, leave-one-out, and 70:30 train-test splits, columns 3,4 and 5 Table S3).”*

*S3 Table. Comparison between different validation methods in random forest classification.*

| *Accuracy metrics* | *K-fold* | *Ratio of the dataset (70:30)* | *Leave-one-out* | *Held-one-year-out* |
| --- | --- | --- | --- | --- |
| *Average RMSE* | *0.31* | *0.28* | *0.22* | *0.31* |
| *Average R2* | *0.57* | *0.56* | *0.57* | *0.50* |
| *Average MSE* | *0.098* | *0.08* | *0.09* | *0.10* |
| *Average percentage RMSE* | *8.08* | *8.10* | *5.91* | *8.10* |

**Reviewer 2**

**Reviewer 2#: The paper estimates rice yield using remote sensing and machine learning models. The topic is interesting and covers an important issue of food security. However, this paper lacks novelty. I have some suggestions which may be addressed before its quality improvement. My comments are below:**

**1. The title is somewhat misleading. Random forest is a very commonly used in crop science.**

Thanks for bringing this up we have modified the title which now read as….*Advancing Food Security: Rice Yield Estimation Framework using Time-Series Optical Data & Machine Learning*

**2. In the abstract, what is the gaps in earlier literature? what is the implication of this research.? The abstract might be comprehensively revised**

We have modified the abstract (changes in Italics), which now reads as:

Lines 15-36 "Timely and accurate rice yield estimation is crucial for supporting food security management, agricultural policy development, and climate change adaptation in rice-producing countries such as Bangladesh. *However, a significant gap exists in the application of remote sensing methods for government-reported rice yield estimation for food security management at high spatial resolution. Current methods are limited to specific regions and primarily used for research, lacking integration into national reporting systems. Additionally, there is no consistent yearly boro rice yield map at a sub-district scale, hindering localized agricultural decision-making.*

To address this need, this study introduced a workflow to enable timely and precise rice yield estimation at a sub-district scale (1,000-meter spatial resolution). This workflow leveraged MODIS and annual district-level yield data to train a random forest model for estimating *boro* rice yields from 2002 to 2021. The results revealed a mean percentage root mean square error (RMSE) of 8.07% and 12.96% when validation was conducted using reported district yields and crop-cut yield data, respectively. Additionally, the estimated yield of *boro* rice varies with an uncertainty range between 0.40 and 0.45 tons per hectare across Bangladesh.

*Furthermore, a trend analysis was performed on the estimated boro rice yield data from 2002 to 2021 using the modified Mann-Kendall trend test with a 95% confidence interval (p < 0.05). In Bangladesh, 23% of the rice area shows an increasing trend in boro rice yield, 0.11% shows a decreasing trend, and 76.51% shows no trend.*

Given that this is the first attempt to estimate *boro* rice yield at a 1,000-meter spatial resolution over two decades in Bangladesh, the estimated mid-season *boro* rice yield estimates are scalable across space and time, offering significant potential for strengthening food security management in Bangladesh. Furthermore, the proposed workflow can be easily applied to estimate rice yields in other regions worldwide.

Keywords: *Boro* rice yield, MODIS, Random Forest."

**3. Introduction section – weak rational justification. How this study can contribute in the literature? What is the hypothesis? These are not clear. There are some related works that used Random Forest model, for example, Ghose et al. 2021.**

**We have cited the article suggested** and the revised sentence now reads in Lines 56-57” *Ghose et al. (2021) food that an increase in temperature by 1°C and rainfall by 1% results in a decrease in Aman rice yields by 33.59% and 3.37%, respectively.****”***

The hypothesis is given in lines 47 - 65 “In recent years, Bangladesh has experienced adverse effects on rice yields due to climate change, characterized by increased extreme weather events, such as droughts and floods during critical phases of rice cultivation [(9)](https://www.zotero.org/google-docs/?tpZqxB). This impact is attributed to the strong dependence of rice yields on weather conditions throughout the cultivation stages [(10)](https://www.zotero.org/google-docs/?FqgE5M). Moreover, Bangladesh's high vulnerability to climate change, reflected in its seventh-place ranking on the Global Climate Risk Index, raises significant concerns, especially in agriculture and rice production [(11)](https://www.zotero.org/google-docs/?3qh6ZR). Moreover, Sarker et al., predicted that between 2005 and 2050, rice production in Bangladesh will decline by an average of 7.4% every year due to climate change [(12)](https://www.zotero.org/google-docs/?CLscfA). Likewise, the National Adaptation Plan of Bangladesh (2030-2050) published by the Ministry of Environment, Forest and Climate Change (MEFCC), Bangladesh [(13)](https://www.zotero.org/google-docs/?lmOBjc), along with the findings from Kaur el al., highlighted the looming threat posed to this crucial agricultural sector, particularly rice, by the escalating impacts of climate change [(14)](https://www.zotero.org/google-docs/?0QXfLH). Ghose et al. (2021) recently revealed that an increase in temperature by 1°C and rainfall by 1% results in a decrease in Aman rice yields by 33.59% and 3.37%, respectively.

The country's vulnerability to these climatic challenges poses a potential threat to food security. It heightens Bangladesh's susceptibility, impacting livelihoods, economic stability, and food security at both national and global levels. This will also pose a significant obstacle to the United Nations Sustainable Development Goals (SDGs), prioritizing achieving zero hunger and promoting sustainable agriculture by 2030 [(12)](https://www.zotero.org/google-docs/?2WtMjf). To manage food security, it is therefore important to accurately estimate rice yields, production, and trends, which could also significantly contribute to efforts to lessen climate-related risks to rice production in Bangladesh. Hence, accurate and timely statistics on rice yields, and long-term trends could assist the government of Bangladesh in managing food security and contribute to efforts to lessen climate-related risks to rice production in Bangladesh.”

Lines 82-97 Similar remote sensing methods are utilized in Bangladesh to estimate rice yield, employing spectral bands and vegetation indices from optical and SAR images (37–39). These methods also include regression-based approaches (40), ML models (41), and deep learning (DL) models (42). However, these methods are primarily utilized for research purposes and need to be integrated into official reporting procedures. In addition, the rice yield maps developed from these studies need to be updated, and their coverage is limited to particular geographic regions within the country. Moreover, the rice yield estimation is often conducted at a coarse scale, typically encompassing districts, divisions, or the entire nation, thus needing more spatial and temporal precision. Currently, annual boro rice yield statistics are available from inventory data at the district level; however, no consistent yearly boro rice yield map is available for Bangladesh at a subdistrict spatial scale, such as a 1,000-meter spatial resolution. Due to this limitation, rice trend analyses conducted in other studies (9,43) are typically done at district and national scales, rendering them less useful for planning and implementing policies at the micro-scale (sub-district level).

This study aimed to evaluate the potential of MODIS data and a random forest (RF) ML-based method to develop a rice yield model for estimating boro rice production in Bangladesh from 2021 to 2022 at a spatial resolution of 1,000 meters. Specific objectives were to 1) evaluate RF models for estimating rice yield in Bangladesh, 2) report the accuracy and uncertainty of boro rice yield estimation by RF models, and 3) understand the spatial and temporal trends of rice yield in Bangladesh from 2002 to 2021.

**How this study can contribute in the literature?**

**We clarify that the higher resolution (1000m) *boro* rice yield estimates compared to existing district-level yield estimates are the most important contribution of this study to the existing body of literature.**

**Line 84 - 97** “ However, these methods are primarily utilized for research purposes and need to be integrated into official reporting procedures. In addition, the rice yield maps developed from these studies need to be updated, and their coverage is limited to particular geographic regions within the country. Moreover, the rice yield estimation is often conducted at a coarse scale, typically encompassing districts, divisions, or the entire nation, thus needing more spatial and temporal precision. Currently, annual *boro* rice yield statistics are available from inventory data at the district level; however, no consistent yearly *boro* rice yield map is available for Bangladesh at a subdistrict spatial scale, such as a 1,000-meter spatial resolution. Due to this limitation, rice trend analyses conducted in other studies [(9,42)](https://www.zotero.org/google-docs/?HAfCtT) are typically done at district and national scales, rendering them less useful for planning and implementing policies at the micro-scale (sub-district level).”

**4. In section 2.2, main weakness of the paper is data quality control. The crop yield data was collected during the years 2019-2020 (n= 2,946) and 2020-2021 (n=1845), specifically focusing on five districts (Dinajpur, Rajshahi, Khulna, Jashore and Rangpur) within Bangladesh. Why only five districts were considered for rice yield analysis for this paper?**

We used district-level reported rice yield data from all districts in Bangladesh from 2006 to 2021 to train and validate our model. Bangladesh Bureau of Statistics (BBS) reports this dataset based on manual surveys. Additionally, we used high-resolution crop yield data from five districts (Dinajpur, Rajshahi, Khulna, Jashore, and Rangpur) within Bangladesh, obtained by manually harvesting the crop and measuring yield directly and scientifically within farmers’ fields. These districts were selected because we had staff and resources available to undertake an assessment of this scope. Unfortunately, other districts were out of scope for grant funds to permit additional crop yield estimates. Collecting crop yield data from the field is time-consuming, resource-intensive, and costly, making it challenging to gather data from all districts.

It is important to note that this crop-cut yield data serves as a secondary source of validation, while the primary source remains the district-level yield data collected between 2006 and 2021 from all districts of Bangladesh. Additionally, other studies also use district-level yield data for training and validation and conduct field surveys in specific districts only to validate satellite-based estimates with measured ground yields[(1–3)](https://www.zotero.org/google-docs/?mzoKfg).

Lines 140 to 144 in the revised manuscript read *“The crop yield data was collected during the years 2019-2020 (n= 2,946) and 2020-2021 (n=1845), specifically focusing on five districts (Dinajpur, Rajshahi, Khulna, Jashore and Rangpur) within Bangladesh (Fig 1b). These districts were chosen due to the availability of qualified scientific staff in these districts that could facilitate an ambitious yield measurement program from farmers’ fields.* Unfortunately, other districts were out of scope for grant funds to permit additional crop yield estimates.*.”*

Line 354-358 “In this study, boro rice crop yield data was collected from five districts in Bangladesh—Dinajpur, Rajshahi, Khulna, Jashore, and Rangpur (Fig. 1b)—due to the availability of data in these regions. Data from other districts were beyond the scope of the grant funding and therefore could not be included. However, in the future, data from additional districts could be collected and used for validation in a similar manner.

*”*

[Islam D, Di L, Qamer FM, Shrestha S, Guo L, Lin L, et al. Rapid Rice Yield Estimation Using Integrated Remote Sensing and Meteorological Data and Machine Learning. Remote Sens. 2023 Apr 30;15(9):2374.](https://www.zotero.org/google-docs/?0j8teK)

[Arumugam P, Chemura A, Schauberger B, Gornott C. Remote Sensing Based Yield Estimation of Rice (Oryza Sativa L.) Using Gradient Boosted Regression in India. Remote Sens. 2021 Jun 18;13(12):2379.](https://www.zotero.org/google-docs/?0j8teK)

[Cao J, Zhang Z, Tao F, Zhang L, Luo Y, Zhang J, et al. Integrating Multi-Source Data for Rice Yield Prediction across China using Machine Learning and Deep Learning Approaches. Agric For Meteorol. 2021 Feb;297:108275.](https://www.zotero.org/google-docs/?0j8teK)

**5. K-fold valuation is used, but how many k-fold are used for this study? Is there any resample technique used for this research?**

We modified the line 207 - 209 which now reads…… *“However, we also test additional cross-validation techniques like hold-one-year-out, leave-one-out, and 70:30 train-test splits (Table S3). A value of k = 5 was used in the k-fold validation technique, where k denoted the number of subsets into which the dataset was divided* [*(4)*](https://www.zotero.org/google-docs/?YFutSd)***.***

**6. Why authors have used only random forest technique and why not other machine learning techniques for this paper?**

We have added the justification and text between line numbers 180 and 183 which reads as…..*“An RF machine learning model was employed to estimate the boro rice yields (Fig. 3). RF is a supervised ensemble method that leverages a collection of decision trees and utilizes the bagging technique (bootstrap and aggregation). Instead of relying solely on individual decision trees, the RF approach aggregates the results of multiple trees to determine the final output.*

*Line 189-193: We chose the RF model because it has demonstrated its capabilities in crop yield estimation, including rice yield estimation* [(66–68)](https://www.zotero.org/google-docs/?GriwQY) *REF. This is due to the RF model's ability to capture the nonlinear relationships between biophysical parameters (input data) and crop yields more effectively than other ML-based models. Additionally, RF is robust to noise, which is crucial since satellite data and crop yield data often contain noisy information that can lead to inaccurate estimates. Furthermore, RF helps prevent overfitting. These factors make the RF model an ideal choice for crop yield estimation.”*

**7. The discussion section is very poor. Scholarly discussion is required. Authors should add limitations to the paper.**

We would like to draw your attention to Section 4.1 Performance of RF Models for *Boro* Rice Yield Estimation, where we thoroughly discussed the limitations of this study and the sources of errors and uncertainties in the estimation.

Line 322-333: Uncertainty in the boro rice yield estimation varied between 0.40-0.45 tons/hectare (Fig. 8) and could be attributed to multiple factors. Firstly, it may result from errors in the district-level yield data reported by the Bangladesh Bureau of Statistics (BBS) from 2007 to 2021. These yields were utilized in the RF model and obtained through farmer interviews or crop cutting. Secondly, using a one-year agriculture mask, derived from the 2020 ESA land cover map, assumed that all crops planted in agricultural regions were rice. However, other crops were also present, albeit covering less than 10% of the area, and they can influence surface reflectance values, potentially introducing noise into the RF model. Thirdly, interannual variations from 2006 to 2021 in the agriculture mask could also introduce uncertainty into the RF model results. A rice crop calendar was used to divide Bangladesh into fourteen crop zones, each with unique rice sowing, peak, and harvest times. However, it is important to note that there were likely variations in the crop calendar at the district level, leading to differences in the timing of sowing, peak growth, and harvest for rice crops. Therefore, these district-level variations in the rice crop calendar must also be considered when accounting for uncertainty in rice yield estimations.

Similarly, in Section 4.2 "Validation of *Boro* Rice Yields Using Crop Cut Data," we discussed the accuracy and limitations of this study by highlighting the sources of errors.

Line 338 - 358: When the estimated rice yields were validated using district-level crop cut data collected for the years 2019-2020 and 2020-2021, the % RMSE increased from 8.17% to 12.96%. This increase in % RMSE by 4.79% could be attributed to several factors. Firstly, these points might not have been accurately sampled from the correct locations, possibly due to uncertainties during the crop-cut experiments. Additionally, it's important to note that MODIS data and other biophysical parameters have a coarser spatial resolution, leading to mixed pixels within the rice fields (75). Despite the coarser spatial resolution, the effects of mixed pixels would contribute less compared to uncertainties in collecting crop-cut data, as rice fields are homogeneously distributed, with 90% of the plantation being rice in Bangladesh.

Rajshahi and Rangpur exhibited high variability in % RMSE (variation of % RMSE = 2.44%) for the years 2019-2020 and 2020-2021. This variability is primarily due to the different number of samples used during these two years. Variations in sample size, as well as differences in sampling design, can significantly impact the accuracy of satellite-based crop yield estimation (76). For Rajshahi, 884 crop cut samples were used for 2019-2020 and 58 for 2020-2021, and for Rangpur, 839 samples for 2019-2020 and 172 samples for 2020-2021 were used. On the other hand, the districts that have nearly the same number of crop cut samples (average percentage difference in sample points = 25.79%) show less variation (average variation % RMSE = 0.27%) in % RMSE. Khulna is an exception where there was an exceptionally high difference in the number of points between the two years (average percentage difference in sample points = 306%), and the percentage RMSE difference was 0.84% (Fig. 5). Improvements to the field sampling strategy could lead to better satellite-based crop yield estimation (71, 76, 77).

Similarly, in Section 4.3 "Comparison of Rice Yields with Previous Studies," we compared our rice yield estimates with previous studies and discussed the potential reasons for both good and bad accuracy.

We also added a limitation in the Future Work section which we thought was missing. The modified sentence read Lines 429-431 *“In this study, crop-cut data from five districts were utilized for the validation of estimated rice yields. However, following the approach discussed in this study, crop-cut data from other districts could also be collected in the future to enhance the validation process.”*

**8. In conclusion, the authors may discuss how this study will be utilized for policy and planning, especially in the case of future food security.**

The following information is given in the following lines:

Line 396 and 404: “This study can be valuable for the Government of Bangladesh to estimate rice yields for future years. Additionally, the government can use this information to create detailed maps showing areas with increasing or decreasing *boro* rice trends and temperature and rainfall trends. This data can inform decisions to enhance *boro* rice production in the near future and adjust *boro* rice crop calendars to adapt to climate change. Furthermore, the *boro* rice yield map can help identify areas where drought-tolerant rice varieties could be introduced to boost *boro* rice production. Moreover, the Bangladesh Bureau of Statistics (BBS) could integrate this innovative rice mapping framework into their conventional *boro* rice yield estimation system to develop an automated national rice yield estimation and monitoring system, thereby contributing to the advancement of smart agriculture in Bangladesh.”

Line 414-420: “Additionally, the *boro* rice yield trend observed from 2002 to 2021 in this study provided valuable insights into areas with decreasing and increasing yield trends. Furthermore, maps depicting rainfall and temperature trends for other studies could help identify regions where *boro* rice yields are currently affected and pinpoint areas where rising temperatures and rainfall trends may negatively affect future *boro* rice yields. The methodology can provide independent, evidence-based information on *boro* rice yield estimates (winter rice), which is useful for stakeholders in Bangladesh for planning and implementing present and future policies related to food security management.”

**References**

[1.	Islam D, Di L, Qamer FM, Shrestha S, Guo L, Lin L, et al. Rapid Rice Yield Estimation Using Integrated Remote Sensing and Meteorological Data and Machine Learning. Remote Sens. 2023 Apr 30;15(9):2374.](https://www.zotero.org/google-docs/?0j8teK)

[2.	Arumugam P, Chemura A, Schauberger B, Gornott C. Remote Sensing Based Yield Estimation of Rice (Oryza Sativa L.) Using Gradient Boosted Regression in India. Remote Sens. 2021 Jun 18;13(12):2379.](https://www.zotero.org/google-docs/?0j8teK)

[3.	Cao J, Zhang Z, Tao F, Zhang L, Luo Y, Zhang J, et al. Integrating Multi-Source Data for Rice Yield Prediction across China using Machine Learning and Deep Learning Approaches. Agric For Meteorol. 2021 Feb;297:108275.](https://www.zotero.org/google-docs/?0j8teK)

[4.	Nti IK, Nyarko-Boateng O, Aning J. Performance of Machine Learning Algorithms with Different K Values in K-fold CrossValidation. Int J Inf Technol Comput Sci. 2021 Dec 8;13(6):61–71.](https://www.zotero.org/google-docs/?0j8teK)
